# New Treatment for Colles Fracture

**Published:** 1870-04

**Authors:** 


### New Treatment for Colles Fracture.

The Medical *Record*, for April 1st, contains Prof. E. M. Moore's paper, read before the State Medical Society at its last meeting, entitled, "A new treatment for Colles Fracture of the Radius, with remarks upon a New Luxation of the Ulnar." The paper we understand received marked attention at the meeting of the State Medical Society, and receives very favorable consideration by the Profession everywhere. The article is illustrated by cuts and is worthy of careful perusal.

